# The first complete mitochondrial genome sequence from the blue-headed parrot (*Pionus menstruus menstruus*): a representative for the genus

**DOI:** 10.1080/23802359.2016.1258341

**Published:** 2016-11-22

**Authors:** Adam Dawid Urantówka, Paweł Mackiewicz

**Affiliations:** a Department of Genetics, Wroclaw University of Environmental and Life Sciences, Wroclaw, Poland;; b Department of Genomics Faculty of Biotechnology, University of Wrocław, Wrocław, Poland

**Keywords:** Androglossini, blue-headed parrot, mitochondrial genome, *Pionus menstruus*, Psittaciformes

## Abstract

*Androglossini* is one of four tribes recognized within a neotropical parrot subfamily *Arinae*. The tribe includes 10 genera of which *Pionus* is represented by eight species. However, its evolutionary diversification and relationship with other *Androglossini* members are still unclear. Depending on studied molecular markers, *Pionus* is closely related with *Amazona* genus or two monotypic genera *Alipiopsitta* and *Graydidascalus* or the clade in which *Amazona* genus is sister to *Alipiopsitta* and *Graydidascalus*. Therefore, we sequenced *Pionus menstruus menstruus* mitogenome to gain molecular data appropriate for future studies to resolve these discrepancies obtained in various phylogenetic analyses published so far.

Neotropical parrots are classified into *Arinae* subfamily, in which four tribes were recognized (Schodde et al. 2013). One of them is *Androglossini*, which includes 10 genera with *Pionus* represented by eight species: *chalcopterus*, *fuscus*, *maximiliani*, *menstruus*, *senilis*, *seniloides*, *sordidus*, and *tumultuosus* (Forshaw 2010). Four of them are monotypic but *chalcopterus*, *menstruus*, *maximiliani*, and *sordidus* are divided into two, three, four, and six subspecies, respectively (Ribas et al. 2007). Most of *Pionus* taxa are distributed in South America except for Central American *P. senilis* as well as *P. menstruus rubrigularis*, which inhabit a region from Costa Rica to Ecuador.

So far, Ribas et al. (2007) presented the most comprehensive study of *Pionus* including 257 specimens of all known subspecies. Their analyses showed the influence of Andes uplift and Pleistocene climatic oscillations on phylogenetic differentiation and biogeographic disjunctions of montane lineages and taxa inhabiting lowland dry and wet forests. However, their molecular analyses did not completely clarify evolutionary history of this genus. Different phylogenetic methods based on two concatenated mitochondrial sequences from cytochrome b and NADH dehydrogenase 2 produced contradictory tree topologies and many internal branches obtained weak or no statistical support. One of these inconsistencies concerns the placement of monotypic species *P. fuscus*, which is located basally to other *Pionus* species or within the tree.

Moreover, the evolutionary relationship of the whole genus *Pionus* with other *Androglossini* members also raises a lot of doubts based on the phylogenies published so far. Depending on the usage of molecular markers, *Pionus* is related with two monotypic genera, *Alipiopsitta* and *Graydidascalus* (Schweizer et al. 2014; Urantówka et al. 2014) or *Amazona* genus (Kirchman et al. 2012) or the clade in which *Amazona* genus is sister to both *Alipiopsitta* and *Graydidascalus* genera (Ribas et al. 2007) or *Graydidascalus* (Schirtzinger et al. 2012).

These controversies imply that more molecular data are required to reconstruct precise phylogeny of *Pionus*. Since it was shown that complete mitochondrial genomes can provide useful information for evolutionary studies of many taxa (Nabholz et al. 2013) and no complete *Pionus* mitogenome is available, we sequenced the mitogenome from *P. menstruus menstruus* to gain appropriate molecular data for future examination of *Pionus* diversification and its relationship to other *Androglossini* members. This subspecies is one of three from *P. menstruus* species and has the most widespread distribution in Amazonian region. The range of isolated *reichenowi* subspecies is limited to southeastern Brazil’s South Atlantic coast, whereas *rubrigularis* also has a disjunctive population in northwestern South America.

The sequence of *P. menstruus menstruus* genome with the length of 18,545 bp was deposited in GenBank under the accession number KX925978. Although morphology of the analyzed specimen (Polish captive bird) was absolutely typical for other *P. menstruus menstruus* individuals, we proved its taxonomic affiliation in phylogenetic analyses of *nd2* sequences including all available *Pionus* taxa with confirmed geographic origins. The obtained tree (Figure 1) revealed that the analyzed individual grouped significantly with another representative of its subspecies. This group was sister to the clade with subspecies *rubrigularis* and *reichenowi*.

**Figure 1.**
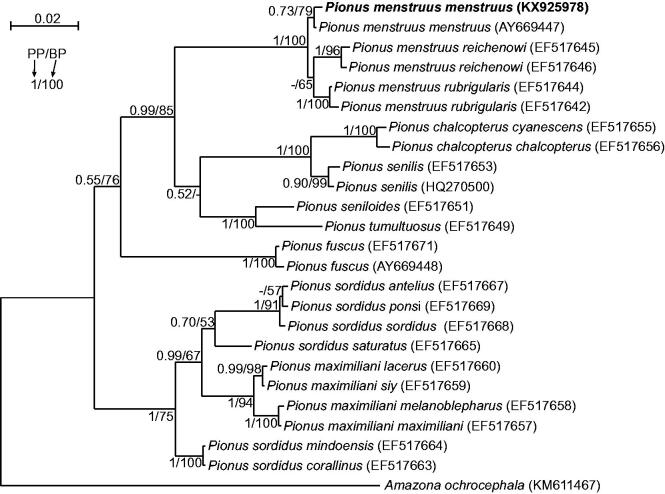
The phylogenetic tree obtained in MrBayes for *nd2* gene indicating that the studied individual (bolded) belongs to *P. menstruus menstruus*. The parrot is kept in aviculture and its blood sample from which DNA was isolated is available in the laboratory at the Department of Genetics in Wroclaw University of Environmental and Life Sciences under the number ADUAKPM02. All *P. menstruus* subspecies create a significant monophyletic clade but *P. sordidus* subspecies are separated by the clade of *P. maximiliani*. It implies that *P. sordidus* is a polyphyletic taxon and should be revised. Values at nodes, in the order shown, indicate posterior probabilities found in MrBayes (PP) and bootstrap percentages calculated in TreeFinder (BP). In the MrBayes (Ronquist et al. 2012) analysis, separate mixed substitution models were assumed for three codon positions with information about heterogeneity rate across sites as proposed by PartitionFinder (Lanfear et al. 2012). We applied two independent runs, each using four Markov chains. Trees were sampled every 100 generations for 10,000,000 generations. After obtaining the convergence, trees from the last 4,717,000 generations were collected to compute the posterior consensus. In the case of TreeFinder (Jobb et al. 2004), the separate substitution models were selected for three codon positions according to Propose Model module in this program, and 1000 replicates were assumed in the bootstrap analysis. The posterior probabilities <0.5 and bootstrap percentages <50 were marked by a dash ‘-’.
